# PCH-2 regulates *Caenorhabditis elegans* lifespan

**DOI:** 10.18632/aging.100713

**Published:** 2015-01-15

**Authors:** Hong Qian, Xiangru Xu, Laura E Niklason

**Affiliations:** ^1^ Department of Anesthesiology, Yale University School of Medicine, New Haven, CT 06520, USA; ^2^ Department of Biomedical Engineering, Yale University, New Haven, CT 06520, USA

**Keywords:** longevity, lifespan extension, sirtuins, stressors, *C. elegans*

## Abstract

Components or downstream targets of many signaling pathways such as Insulin/IGF-1 and TOR, as well as genes involved in cellular metabolism and bioenergetics can extend worm lifespan 20% or more. The *C. elegans* gene *pch-2* and its homologs, including *TRIP13* in humans, have been studied for their functions in cell mitosis and meiosis, but have never been implicated in lifespan regulation. Here we show that over-expression of TRIP13 in human fibroblasts confers resistance to environmental stressors such as UV radiation and oxidative stress. Furthermore, *pch-2* overexpression in *C. elegans* extends worm lifespan, and enhances worm survival in response to various stressors. Conversely, reducing *pch-2* expression with RNAi shortens worm lifespan. Additional genetic epistasis analysis indicates that the molecular mechanism of *pch-2* in worm longevity is tied to functions of the sirtuin family, implying that *pch-2* is another chromatin regulator for worm longevity. These findings suggest a novel function of the *pch-2* gene involved in lifespan determination.

## INTRODUCTION

The Pachytene CHeckpoint 2 (*pch-2*) gene has been studied for its functions in monitoring and correcting DNA errors during cell mitosis [1]. In budding yeast, *PCH2* and silent information regulator 2 (*SIR2*) are found predominantly in the nucleolus. Mutation of *PCH2*, or *SIR2*, bypasses checkpoint-induced pachytene arrest, and *PCH2* and *SIR2* are both needed to prevent meiotic interhomolog recombination within the repeated ribosomal RNA genes that are present in the nucleolus [1, 2]. Increased expression of the *SIR2* family proteins, now called the ‘‘sirtuins,’’ has been shown to enhance lifespan in a range of organisms, including *S. cerevisiae*, *C. elegans*, *D. melanogaster*, and *M. musculus*. The lifespan extension for animals imparted by the sirtuins is up to 50% [3]. However, new data indicate that some of the reported effects in *C. elegans* and *D. melanogaster* may have been due to confounding genetic backgrounds and other aspects in experimental designs [4-6]. The latest study in brain-specific *Sirt1*-overexpressing (BRASTO) transgenic mice demonstrated a significant life span extension of 11%, and aged BRASTO mice also exhibited a clear delay in aging phenotypes [7]. Over-expression of *Sirt6*, another mammalian homolog of *Sirt2*, extends lifespan in male mice around 15%, but has no effects in females [8]. However, no functional association between *PCH2* and *SIR2* has ever been linked to aging or to lifespan before. Based on these observations, we investigated whether there exists a link between *pch-2* expression, and lifespan, in *C. elegans*. Our findings suggest that there is an association.

## RESULTS

**Figure 1:**
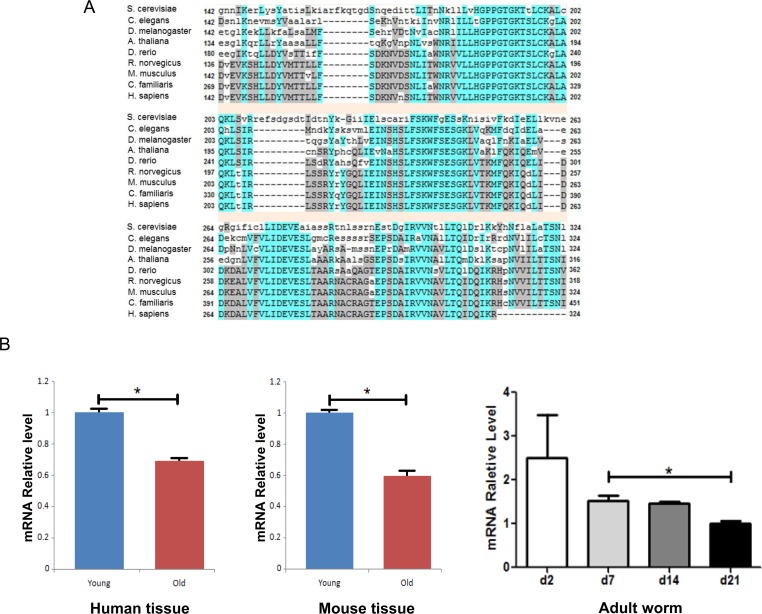
Pch-2 is an evolutionarily conserved gene and its expression declines with tissue aging across species (**A**) Sequences alignment of pch-2 genes cross species ranging from yeast, worm, fly and rodent, to human. (**B**) Pch-2 mRNA expression changes with age in human, mouse and worm. * indicates *p* <0.05.

To study the evolutionary conservation of *pch-2*, we first examined protein sequences of Pch-2 and its homologs among various species using the NCBI RefSeq database. We performed a phylogenetic analysis using Phylogeny.fr [9], and the protein sequence alignment of Pch-2 and its homologs was evaluated. Pch-2, similar to Sir2, is an evolutionarily conserved gene with a functional domain of P-loop_NTPase, that is found across species including yeast (*S. cerevisiae*), worm (*C. elegans*), fly (*D. melanogaster*), zebrafish (*D. rerio*), rodent (*R. novegicus*, *M. musculus*) and human (*H. sapiens*) (Fig. 1a & Supplement Table 1). Interestingly, the meiotic checkpoint function of *pch-2* and its homologs have been studied not only in yeast, but also in worm and mouse [10, 11]. Collectively, these findings provide evidence for evolutionarily conserved functions for *pch-2* in both recombination, and in the formation of higher order chromosome structures. We thus concluded that *pch-2* provides some fundamental function across the animal kingdom.

To evaluate expression of *pch-2* homologs in mammalian species, we examined brain and gonadal tissues from mouse and human samples, since *pch-2* is known to be involved in preventing meiotic inter-homolog recombination. We measured expression levels of *pch-2* homolog *TRIP13* mRNA in young subjects (N=5 for 3 month-old mice from two aging colonies, C57B/6 and DBA2; N=8 for 18-25 year-old humans) and old subjects (N=5 for 22 month-old for mice, from two aging colonies; and N=10 for >60 year-old humans). We found that expression declines significantly with physiological aging in ovary and brain tissues of both human and mouse (Fig. 1B). This was judged by both fold change in mRNA expression, as well as by *p*-values for mouse and human tissues. We also observed a clear trend of decreasing *pch-2* expression in *C. elegans* over the lifespan of the animals, from day 2 to day 21 (Fig 1B). This observation suggests an evolutionarily conserved loss of expression of *pch-2* with tissue aging, in *C. elegans,* mouse and human.

**Figure 2:**
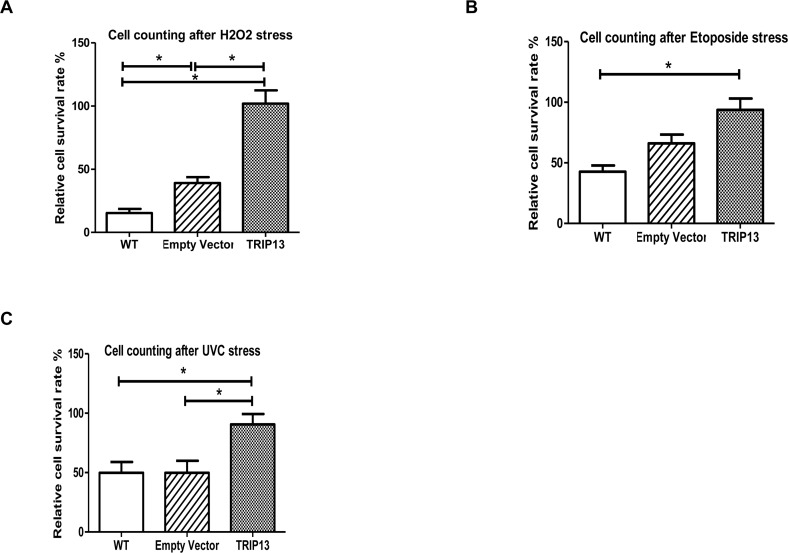
TRIP13 over-expression human fibroblast cells are resistant to stressors of oxidation, apoptosis and DNA damage **(A)** After 600uM H_2_O_2_ treatment, the TRIP13 over-expression fibroblast cells showed enhanced cell numbers when compared to control cells, wild type (WT) and empty vector (*p*<0.0001). **(B)** TRIP13 over-expression fibroblasts were treated with 10uM etoposide for 3 days, and exhibited higher cell numbers following treatment (*p*<0.001). **(C)** TRIP13 cells demonstrated significantly higher resistance after UV radiation when compared to the control groups (*p*<0.01). (Relative cell survival was as compared to numbers of cells plated originally, prior to culturing and stressors.) * indicates significant differences between groups.

To investigate any potential role in the *pch-2* gene family in lifespan and stress resistance, we first generated a human *pch-2* homolog - *TRIP13* - over-expression retroviral vector (see Supplement). We used this retrovirus to infect normal dermal human fibroblasts under hygromycin selection, and compared the TRIP13-overexpressing fibroblasts to those infected with empty vector, as well as non-infected controls. We found, after retroviral infection and hygromycin selection, that doubling times of WT, empty vector, and TRIP13 over-expressing populations were 57 hour, 84 hours and 130 hours, respectively. After populations were established under selection, we applied the environmental stressors 600uM H_2_O_2_ (oxidative stress), 10uM etoposide (apoptotic stress) and the UV radiation (DNA damage stressor) at 50J/m2. After 600uM H_2_O_2_ treatment, the TRIP13 over-expressing populations showed significantly enhanced cell survival rate when compared to control groups, both the empty vector and WT (Fig. 2A, p<0.0001). When treated with 10uM etoposide for 3 days, TRIP13 over-expressing cells also survived at a significantly higher rate (Fig. 2B, p<0.001). Similar results were observed in TRIP13 over-expressing populations after UV radiation when compared to the control groups (Fig. 2C, p<0.01). Importantly, since the population doubling times of TRIP13-transfected cells was slower than that of controls, this improved survival after application of stressors was not simply an artifact of faster cell cycling. This indicates that TRIP13 confers an enhancement of fibroblast cell survival under various stressors of oxidation, apoptosis and DNA damage. All of these stressors are potentially involved in the normal aging process.

**Figure 3:**
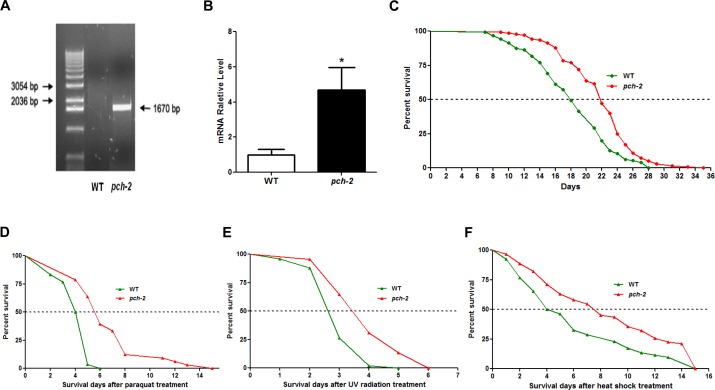
*Pch-2* over-expression extends lifespan and enhances stress-resistance in *C. elegans* (**A**) genotyping of *pch-2* shows a band at 1670bp indicating the transgene in the *pch-2* over-expressing line. (**B**) qRT-PCR validation of *pch-2* over-expression worm lines by using transgene-specific primers shows increased *pch-2* gene expression (*p*<0.01). (**C**) lifespan measurement was conducted for both WT (GFP-expressing, n=126) and *pch-2* over-expressing (n=140) animals. Both median lifespan and maximum lifespan of *pch-2* over-expression lines show a 25% extension when compared to WT lines (*p*<0.001, *p* values were derived from student t test and log-rank test). (**D**) After 4mM paraquat treatment, the median survival of *pch-2* over-expressing lines (n=33) was increased by 40%, and the maximum survival of *pch-2* over-expressing lines was increased 150% (n=30) (*p*<0.001). (**E**) The median survival of *pch-2* over-expressing lines (n=49) was increased 45% after UV radiation when compared to WT (n=45) (*p*<0.001). (**F**) With heat shock, the median survival of *pch-2* over-expressing lines (n=62) was increased by 85% when compared to WT (n=52) (*p*<0.001).

Based upon these observations, we wondered whether *pch-2* plays a role in longevity. Given that lifespan in *C. elegans* is only 3-4 weeks, as opposed to approximately 2-3 years in mice [12, 13], we elected to examine the impact of the *pch-2* gene on lifespan in *C. elegans*. We first generated *pch-2* over-expressing *C. elegans* lines by a microinjection method (Supplement Fig.1), and studied the functional impact of *pch-2* on worm longevity. To identify over-expressing *pch-2* lines, we selected those animals co-expressing GFP by direct visualization. In GFP-expressing lines, we also measured the expression levels of *pch-2* using quantitative reverse transcription polymerase reaction (qRT-PCR). We distinguished between the expression of endogeneous and exogenous *pch-2* by using an expression vector-specific primer and a *pch-2*-specific primer. In GFP-positive over-expressing lines, the level of exogenous *pch-2* expression was three times higher than the level of endogenous *pch-2* in the wild-type (WT) controls with selection marker GFP (Fig. 3A, Fig. 3B). After confirming over-expression of *pch-2*, we then subjected *pch-2* over-expressing and WT worms to a lifespan measurement [14]. The median lifespans of WT and *pch-2* over-expressers were 18 days and 22 days, respectively, with maximum lifespans of 28 days and 35 days, respectively (Fig. 3C). Hence, *pch-2* over-expressing lines extended both median and maximum lifespan by approximately 25% as compared to WT (log rank test, n=126/140, *p*<0.001).

We next determined whether *pch-2* over-expressing lines could withstand various stressors better than WT (GFP-expressing) controls. Young adult worms with *pch-2* over-expression and controls were treated on day 1 with the oxidative stressor paraquat at 4 mM for their survival duration. The median survival of *pch-2* over-expressing lines was 5.6 days, while that of control lines was 4.0 days. The maximum survival of *pch-2* over-expression and WT lines were 15 days and 6 days, respectively, which is an increase of 40% and 150% in median and maximal survival for *pch-2* over-expressers (*p*<0.001 for both median and maximal survival as compared to WT controls by log rank test) (Fig. 3D).

To provide a DNA damage stressor, day 1 worms were exposed to UV radiation at 0.1 J/cm2. The median survival of *pch-2* over-expressing lines was 3.4 days and that of control lines was 2.6 days after UV radiation, while the maximum survival were 6 days and 5 days, respectively (45% and 20% higher for *pch-2*, respectively, *p*<0.001) (Fig. 3E). Lastly, heat shock was administered on day 1 worms at 35°C for 2 hours, after which animals were removed to routine conditions and their survival tallied. Intriguingly, the median survival of *pch-2* over-expression lines was 85.0% longer than that of control lines (7.4 days vs. 4 days) (*p*<0.0001), but the maximum survival was not changed between two lines (Fig. 3F). Hence, *pch-2* conferred significantly increased resistance to multiple stressors which affect both DNA and protein integrity [15].

**Figure 4:**
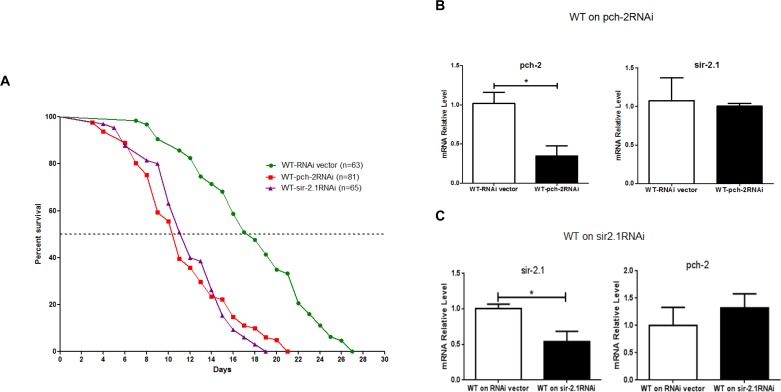
Inhibition of *pch-2* and *sir-2* expression by RNAi impacts lifespan (**A**) A shortened lifespan, both median (62%, 64%) and maximum (70%, 78%), was caused by *pch-2* and *sir2* specific RNAi (n=81, 65) as compared to RNAi vector lines (n=63) (*p* <0.0001). (**B**) In WT worms, RNAi for *pch-2* resulted in significantly reduced expression by ~ 70% as validated by qRT-PCR, but did not have a significant effect on *sir-2* transcripts. (**C**) Similarly, in WT worms, RNAi for *sir-2* resulted a significant reduction of *sir-2* expression about 50% by qRT-PCR validation, and without notable effect on *pch-2* expression (*p <0.001).

As further confirmation of our observations, we examined lifespan after RNAi-induced *pch-2* and *sir-2* knockdown in WT N2 animals. A *pch-2* specific RNAi clone F10B5.5 and a *sir-2* specific RNAi clone T27E4.8 were used to feed WT N2 worms, thereby reducing *pch-2* and *sir-2* expression. The median lifespan of RNAi vector animals was 17 days, but this was shortened to 10.5 days after *pch-2* RNAi treatment. Maximum lifespan shortened to 19 days from 27 days, and both median and maximum lifespan were significantly shortened by *pch-2* RNAi treatment (*p*<0.0001) (Fig. 4A). Interestingly, similar results were also obtained from RNAi inhibition in *sir-2* WT N2 animals (Fig. 4A). Only one-third expression level of *pch-2* and *sir-2* remained after RNAi treatment, as assessed by qRT-PCR (Fig. 4B & C). Furthermore, RNAi treatment was specific, and treatment of worms with *pch-2* RNAi did not impact transcript levels for *sir-2*, for example. Hence, knockdown of *sir-2* and *pch-2* resulted in similar decrements in lifespan in WT worms.

**Figure 5:**
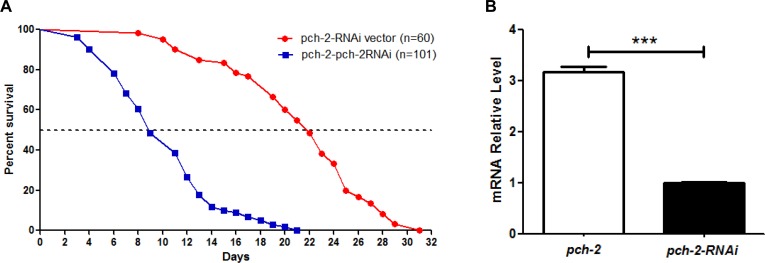
Inhibition of *pch-2* by RNAi impacts lifespan of *pch-2* over-expressing *C. elegans* (**A**) A shortened lifespan, both median (30%) and maximum (56%), was induced by *pch-2*-specific RNAi in *C. elegans* that were engineered to over-express *pch-2* (n=101), as compared to RNAi vector lines (n=60) (*p* <0.0001). (**B**) qRT-PCR validated the RNAi effect, indicating a significant reduction (~70%) of *pch-2* transcripts in *pch-2* over-expressing worms treated with RNAi. ***p <0.001.

In addition, we evaluated the lifespan of *pch-2* over-expression worm lines when subjected to *pch-2* RNAi treatment. The median lifespans of *pch-2* RNAi and *pch-2* over-expressers were 7 days and 23 days, respectively, with maximum lifespans of 20 days and 36 days, respectively (Fig. 5). Hence, *pch-2* RNAi inhibition in *pch-2* over-expressing lines shortened both median and maximum lifespan significantly when compared to *pch-2* over-expressing worms (log rank test, *p*<0.0001).

**Figure 6:**
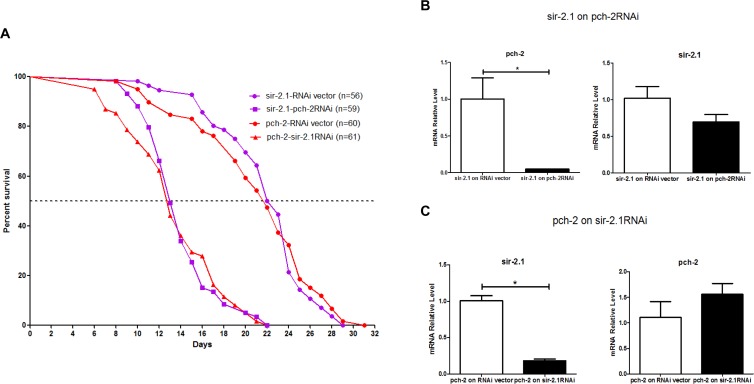
RNAi inhibition of both pch-2 in sir-2.1 over-expressing worms, and sir-2.1 in pch-2 over-expressing worms, shortens lifespan (**A**) Purple curves show lifespans of *sir-2.1* over-expressing worms, while red curves show lifespans of *pch-2* over-expressing worms. In *sir-2* over-expressing worms, *pch-2* RNAi significantly decreased lifespan (56% and 75% decrease in median and maximum lifespan, respectively). In *pch-2* over-expressing worms, *sir-2.1* RNAi induced a 57% and 70% decrease in median and maximum lifespans (*p* <0.0001 for both effects). (**B**) qRT-PCR validated the RNAi effect, indicating a significant reduction (~90%) of *pch-2* transcipts in *sir-2* over-expressing worms that were treated with *pch-2* RNAi. (**C**) qRT-PCR validating a significant reduction (~70%) of *sir-2* transcripts in *pch-2* over-expressing worms that were treated with *sir-2* RNAi. *p <0.001.

To further investigate the role of *pch-2* in worm longevity, we wondered if *pch-2* and *sir-2* may have functional connections in worm lifespan determination. Therefore, we scrutinized the lifespan of *pch-2* over-expressing worm lines with *sir-2* RNAi treatment, and the *sir-2* longevity worm line (LG394 from CGC) when subjected to *pch-2* RNAi treatment. The median lifespans *sir-2* longevity animals that were subjected to *pch-2* RNAi, and the *sir-2* longevity line itself, were 14 days and 23 days, with maximum lifespans of 22 days and 29 days, respectively (Fig. 6A). The median lifespans of *pch-2* over-expressors that were exposed to *sir-2* RNAi, and the untreated *pch-2* over-expressers, were 14 days and 23 days, with maximum lifespans of 22 days and 31 days, respectively (Fig. 6A). Hence, *pch-2* RNAi inhibition in the *sir-2* longevity line, and *sir-2* RNAi inhibition in *pch-2* over-expressing lines, shortens both median and maximum lifespan significantly, and by a similar amount (log rank test, *p*<0.0001 for both lines) (Fig.6). qRT-PCR confirmation on the impact on the transcript levels of *pch-2* and *sir-2* RNAi showed a clear effects of *pch-2* and *sir-2* inhibition (Fig. 6B & C), confirming that RNAi treatment knocked down the target transcripts. However, there were no observed cross-over effects of both RNAi administrations on each other (Fig. 6B & C). These genetic epistasis studies show that *pch-2* and *sir-2* operate independently and reciprocally to extend lifespan of worms, and that expression levels of one gene do not appear to directly impact expression levels of the other gene.

## DISCUSSION

Classic genetic and functional genomics screens have identified a number of genes that are involved in *C. elegans* lifespan regulation. Dozens of single-gene mutations have been demonstrated to lead to worm lifespan extension of 20% or more. Many of these genes are components or downstream targets of the Insulin/IGF-1 and TOR signaling pathways [12, 16]. Intriguingly, *reducing* the expression of most of these genes by RNAi, for example, *increases* worm lifespan [17]. There are a small proportion of the Insulin/IGF-1 pathway downstream key mediators, such as, DAF-16, SKN-1 and DAF-16-regulated genes including HSF-1 and RPN-6, that extend worm lifespan in over-expressors [18-21]. *Pch-2*, however, is not known to be involved with either the Insulin/IGF-1 or the TOR pathways. In current studies, over-expression of TRIP13, the human homolog of *pch-2*, in human fibroblasts exhibits enhanced resistance to environmental detrimental stressors including oxidative stress, apoptosis and UV radiation. The cells with over-expression Trip13 have a slower proliferating rate than controls, meaning that increased proliferation in Trip13 over-expressors cannot account for observed increases in cell number after stress. Furthermore, over-expression of *pch-2* in *C. elegans* led to increased lifespan, thereby suggesting that *pch-2* is protective against worm aging. Conversely, decreasing *pch-2* expression with RNAi led to a decrease in lifespan. The mechanism by which pch-2 enhances lifespan may be independent of the widely-studied Insulin/IGF-1 and TOR pathways, but may possibly be related to mechanisms by which the sirtuins increase lifespan, given that inhibition of both *pch-2* and *sir-2* decreased lifespan by similar amounts, and given the spatial co-localization of these gene products in nucleoli. Since the expression of *Trip13* declines with mammalian tissue aging (Fig. 1), these data taken together suggest that *pch-2* (mammalian Trip13) may play an important role in the aging process.

**Figure 7:**
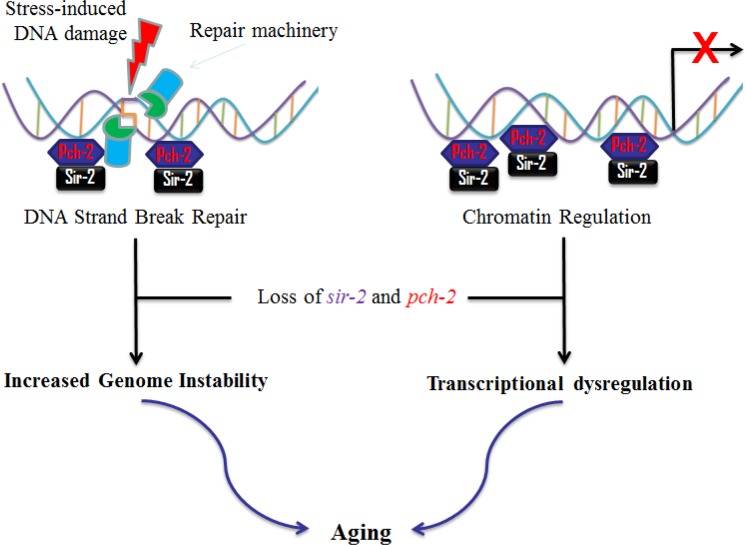
Proposed interactions of *pch-2* and *sir-2* in aging in *C. elegans* Based on our data and the literature, we propose that *pch-2* may, similar to *sir-2*, affect the aging process of *C. elegans*. Briefly, *pch-2* may play roles in surveillance of DNA damage and chromatin regulation, in concert with *sir-2*. Reduction of *pch-2* expression induces genome instability and transcriptional dysregulation, and, therefore, may promote aging in *C. elegans*.

Studies suggest that the anti-aging effects of *sir-2* and the sirtuins are at least partly through maintenance of genome stability, by sensing and repairing damaged DNA. Sirtuins may also be playing a role as chromatin regulators in silencing unfavorable gene transcription [20], and in regulating the metabolic response to environmental stimuli [22]. Both *pch-2* and *sir-2* have been closely associated for checkpoint functions^2^ and protection of repetitive DNA (rDNA) in yeast [23]. Interestingly, our investigations on worm lifespan modulation suggest that *pch-2* and *sir-2* independently and reciprocally influence lifespan. We therefore propose a plausible mechanism that *pch-2* is interacting with *sir-2* in lifespan extension (Fig. 7). However, it is still unclear how *pch-2* and *sir-2* interact to influence downstream effectors, while regulating lifespan in worms and, possibly, in mammals.

In summary, we have described a novel gene that is involved in lifespan extension in *C. elegans.* This gene effectively extends lifespan in over-expressing lines under a variety of stressors, and knockdown of the gene results in shortening of lifespan. The mechanism of action of pch-2 may be tied to the actions of the sirtuin family, though more studies are needed on how they impact functions of each other in terms of lifespan determination. Furthermore, the lifespan effects of this gene should also be studied in other animal systems, including in mammalian species.

## METHODS

### Nematode strains and maintenance

The *C. elegans nematode* strain N2 used for all the experiments was a gift from the Reinke lab. *Sir-2* over-expressing line LG394 was purchased from Caenorhabditis Genetics Center (CGC). All the *C. elegans* stocks, including constructed strains, were maintained at 25 °C on nema-tode growth medium agar (NGM) plates seeded with *E. coli* strain OP50, as described in WormBook [24].

### Molecular cloning

Multiple DNA fragments were cloned by using Gateway three-fragment vector construction kit. (Invitrogen, Carlsbad, CA).

*Step 1*: Produce three fragments with flanking site by PCR:

Three fragments: promoter *myo-3* (gift from Koelle lab), gene GFP or *pch-2*, and 3′-UTR were amplified by PCR using primers that incorporate flanking attB4 and attB1r sites in fragment *myo-3*, flanking attB1 and attB2 sites in the GFP or *pch-2* genes, and flanking attB2r and attB3 sites in 3′-UTR. Templates for amplifying *myo-3*, GFP, and 3′-UTR are others’ plasmids (gift from Reinke lab), and the gene *pch-2* was amplified from *C. elegans* genomic DNA (gift from Reinke lab).

*Step 2*: Entry clones were generated by BP reaction:

Three PCR products from step 1and three donor vectors P4-P1r, P1-P2, P2-P3 were used in three separated BP recombination reactions between an att B-flanked DNA fragment and an att P-containing donor vector to generate an entry clone. We generated three entry clones: pENTR^TM^L4-R1-*myo-3*, pENTR^TM^L1-L2-GFP and pENTR^TM^L1-L2-*pch-2*, and pENTR^TM^R2-L3-3′-UTR.

*Step 3*: Expression clones were generated by LR reaction:

Expression clones were generated by LR reactions between an att L-containing entry clone and an att R-containing destination vector. Three entry clones from step 2 and the destination vector pDEST^TM^R4-R3 were used together in a single LR reaction to generate the expression clones pCFJ150 with 3 fragments, which were named pCFJ150-GFP and pCFJ150-*pch-2*.

### Microinjection

A DNA mixture of 10 ng plasmid pCFJ150-GFP, and 10 ng plasmid pCFJ150-*pch-2*, and 50 ng carrier plasmid pUC-19 was microinjected into the syncytial gonad of wild type N2 animals**^24^**. After 48-72 hr, we scored the progeny of injected worms using a fluorescent stereomicroscope (Olympus S2x16). Each green transformed progeny was transferred to a separate NGM plate as an independent line using a worm pick. Only the lines for which the F1 progeny could pass the transgene onto their progeny with efficiencies (green positive worms as a fraction of all progeny) greater than 50% were kept and used in further experiments. GFP alone with the carrier DNA were injected to obtain the control lines for these experiments. Each transgenic line was maintained by transferring 5-10 green worms to a new NGM plate every 3-4 days.

### Genotyping

Single adult worms were picked and put into 10 ul lysis buffer (50 mM KCl, 10 mM Tris pH8.3, 2.5 mM MgCl2, 0.45% NP40, 0.45% Tween 20, 0.01% gelatin) with fresh 1.0 mg/ml Proteinase K. Worms were digested at 60°C for an hour and 95°C for 15 min. Digested lysate template was amplified by PCR using oligonucleotide primer sequences forward: 5′-ctatgaccatgattacgccaagc; and reverse: 5′-gatgatgaggattcacgacaca. The PCR product was indicated by a 1670bp band on a 1% agarose gel. The *pch-2* over-expressing lines were all genotyped to ensure the over-expressors were really over-expressing *pch-2*.

### Lifespan assay

To quantify lifespan, L4 larvae from the age-synchronized population of worms were transferred to NGM plates supplied with 100ug/ml Ampicillin and 500 nM 5-fluoro-20-deoxyuridine (FUDR) seeded with sufficient OP50 bacteria. Worms were monitored by tapping their head with a platinum worm pick every 1 or 2 days until they were dead. Worms were scored as dead if they did not respond by moving the head to tapping. Worms which had fled or crawled off the agar and died on the side were censored and removed from analysis [25]. At least three individual experiments were performed in each group.

### Paraquat treatment and heat shock

Age-synchronized L4 larvae were first transferred to FUDR plates. After 24 hr, for the paraquat assay young adults were moved to FUDR plates supplied with 4mM paraquat for the duration of the experiment [26]. For heat shock, the plates with young adult worms were moved to a 35°C incubator for 2 hr, and then removed back to 25°C conditions [27]. All of the worms were subsequently monitored every day for survival, and survival curves were based on daily counting.

### UV radiation

Young adult worms were irradiated on NGM plates without OP50 under a germicidal bulb (254 nm) at 0.1 J/cm2 by using an UV crosslinkers. (CL-1000 Ultraviolet Crosslinkers, LLC Upland, CA, US). Then the worms were transferred to FUDR plates that were seeded with OP50. Worms were checked daily through their lives to generate survival curves [17].

### RNAi induction

Gene knock down by RNAi was performed by feeding the worms with bacteria which produced dsRNA against the gene of interest. RNAi for *pch-2* was a gift (Weidhaas lab). Briefly, on the first day, the RNAi clone in *E. coli* was incubated overnight at room temperature on RNAi agar plates with 25 μg/ml carbenicillin and 1 mM isopropylthiogalactosidase (IPTG) to induce dsRNA expression. On the second day, L4 larvae were transferred to the seeded plates to be monitored for their life spans. Bacteria containing RNAi empty vector were used as food for the control group [28]. *Sir-2* over-expressing line LG394 and WT worms were studied with RNAi.

### RNA isolation and Quantitative PCR

Total RNA was isolated from 10 adult worms per sample using RNeasy mini kit from QIAGEN. Nematodes were washed in M9 buffer**^27^** three times and excess M9 was carefully removed. The samples were re-suspended in 350 μL lysis buffer with β-mercaptoethanol, mixed with an equal volume of 70% ethanol. The mixture was transferred to a spin column and followed by washing steps according the manufacturer's protocol. DNase digestion was performed in the column and RNA was eluted in 13 μL RNase-free water. Total of 12 μL RNA was used to synthesize cDNA using the Ominscript kit (QIAGEN). For real-time PCR, each 25 μL reaction containing 12.5 μL of 2x SybrGreen supermix (Bio-Rad), 0.4 μM of each primer, and 2 μL of template cDNA was performed on a C1600 Thermal Cycler (Bio-Rad). Relative gene expression level was normalized to act-1 and calculated using the ΔΔCt (cycle threshold) method.

### Examination of the expression of pch-2 homologs in mouse and human tissues

Total RNA of mouse and human ovaries were isolated with the RNeasy mini kit (QIAGEN). Five young animals (3 months old) and 5 old animals (22 months old) were used for the gene expression analysis. Eight young humans (ages 18-25 yrs old) and 10 old humans (ages >60 yrs old) were included for this study. Gene expression was analyzed by Affymetrix gene array (version ST 1.0) and the differential expression of *pch-2* homologs was judged by both fold change and by *p* value [24].

#### Fibroblast cell culture

Human normal adult dermal fibroblast cells were purchased from ATTC (Manassas, VA). Cell culture was maintained at 37°C, 5% CO2 in DMEM (Invitrogen, Grand Island, NY) supplemented with 10% FBS and 100u/ml Penicillin-Streptomycin.

#### Retroviral vector constructs

Human TRIP13 full length cDNA was amplified from construct pCMV6-XL5-TRIP13 (Origene, Rockville, MD) by PCR using two cloning site tagged primers hTRIP13-1F &hTRIP13-1R (see Table1) and cloned into *SalI* and *BamHI* sites of pBABEhygro-hTERT (Cell Biolabs, San Diego, CA). The insert was confirmed by sequencing. Control empty vector pBABE-hygro was from Addgene. (Cambridge, MA).

#### Transfection and cell infection

Retroviruses were produced by transfection of the retroviral constructs in the Platinum-A Retroviral packing cell line (Cell Biolabs). Briefly, Plat-A cells were grown to 70% confluency in 10-cm dishes with DMEM, 10% FBS without antibiotics and transiently transfected with Lipofectamine (Invitrogen). Purified 10 ug plasmid DNA of pBABEhygro-hTRIP13 was mixed with Lipofectamine 2000 transfection reagent 30ul in Opti-MEM according to the manufacturer's protocol and were incubated overnight, then exchanged with growth medium without antibiotics. After 48 hours of infection, retroviral supernatants were collected by brief centrifugation at 1200 rpm for 5 minutes. Empty vector pBABEhygro was packaged in parallel as control.

Human dermal fibroblast cells were grown to 50% confluency and infected by retrovirus with growth medium at ratio 1:1 for 2 doses of overnight in the presence of 10 ug/ul polybrene. After 72 hours of infection, the infected cells were selected and maintained in growth medium with 90 ug/ml hygromycin to obtain a stable line. Wild type (WT) was uninfected fibroblast cells at the same passage of infected lines as one of the controls. Empty vector cell line was generated by infection with pBABE-hygro without the transgene insert. The TRIP13 cell line was generated by infection with pBABEhygro-TRIP13.

### Cell culture stresses

#### 1) H_2_O_2_ treatment

Cells were plated in 6-well plates for 24 h and replaced by growth medium with 600 uM

H_2_O_2_ and incubated for 2 h. Then cells were changed back with growth medium and incubated for 4 days. Cells were trypsinized and split at a 1:2 ratio in 6-well plates and incubated for 24 h. Cells were treated for a second time H_2_O_2_ for 2 h and then were replaced with growth medium for 24 h.

#### 2) Etoposide treatment

Etoposide (Sigma, St. Louis, MO) was dissolved in Me_2_SO and added to growth medium at final concentration of 10 uM. Cells were plated in 6-well plates for 24 h and replaced by growth medium with etoposide for 3 days. Growth medium add Me_2_SO only was as control.

#### 3) UVC radiation

Cells were plated in 6 well plates for 24 h and replaced medium with PBS. Cells were put in a crosslinker (UVP, Upland, CA) for UVC exposure at 50J/m2. Then cells were changed back into growth medium and incubated for 24 h.

#### 4) Cell counting

Cells were washed with PBS and trypsinized with 0.25% trypsin for 5 minutes. Cells were diluted in growth medium and mix with equal volume of trypan blue, and 20 ul of mixture was loaded into counting chamber and counted by Cellometer Auto T4 Cell Counter. (Nexcelom Bioscience, Lawrence, MA). All cells were counted 24 hours after administration of the final stress for that condition. For all experiments, cell stress was performed on triplicate cultures (n=3).

## SUPPLEMENTARY TABLE AND FIGURES


