# Supplementary material for: PCH-2 regulates Caenorhabditis elegans lifespan
Source: Aging (Albany NY). 2015 Jan 15;7(1):1–11. doi: 10.18632/aging.100713 (PMC4350321; doi:10.18632/aging.100713)
Supplement: Supplementary file 1 [file aging-07-1-s001.pdf]

RNA isolation and Quantitative PCR

Total RNA was isolated from using RNeasy mini kit from QIAGEN (Valencia, CA).

For cells: Cell plates were washed with PBS twice and resuspended in 350 ul lysis buffer with β-mercaptoethanol; For worms: 10 adult worms per sample were washed in M9 buffer for three times and excess M9 was carefully removed. The samples were resuspended in 350 ul lysis buffer with β-mercaptoethanol. Equal volume of 70% ethanol was mixed into the lysis buffer. The mixture was transferred to a spin column and followed by washing steps according the manufacturer protocol. DNase digestion was performed in the column and RNA was eluted in RNase-free water (25 ul in cell samples and 13 ul in worm samples). Total 1ug cell RNA or 12 ul worm RNA was used to synthesis cDNA by using Ominscript kit (QIAGEN). For real-time PCR, each 25 ul reaction containing 12.5 μl of 2x SybrGreen supermix (Bio-Rad), 0.4 μM of each primer hTRIP13-2F&2R;hGAPDH-F&R;pch-2-1F&1R; sir-2.1-F&R; act-1-F&R

(See Table1) and 2 μl of template cDNA was performed on a C1600 Thermal Cycler (Bio-Rad). Relative gene expression level was normalized to GAPDH or act-1 and calculated using the ΔΔCt (cycle threshold) method.

Primers were used for studies:

	**Cloning primers**
hTRIP13-1F	5'-gtattaaggatcctacgtaatggacgaggccgtgg
hTRIP13-1R	5'-acagggtcgactcagatgtaagctgcaag
attB1-gfp-F	5'-ggggacaagtttgtacaaaaaagcaggctcgttcaccatgagtaaaggagaa
attB2-gfp-R	5'-ggggaccactttgtacaagaaagctgggtccagcggccgatgttagttagtta
attB1-pch-2-F	5'-ggggacaagtttgtacaaaaaagcaggctcgtcagactaaagtatgcacgag
attB2-pch-2-R	5'-ggggaccactttgtacaagaaagctgggtctaaaatttaattatttctact
attB4-Pmyo3-F	5’-ggggacaactttgtatagaaaagttgaacggctataataagttctt
attB1r-Pmyo3-R	5’-ggggactgcttttttgtacaaacttgttctagatggatctagtgg
pch-2-R	5'-gatgatgaggattcacgacaca
Pmyo3-F	5'-caaatttctcggcgatttgt
	**mRNA primers**
hTRIP13-2F	5'-tgtgtaaagcgttagcccaga

hTRIP13-2R	5'-gccactttccgaaaaccactta
hGAPDH-F	5'-ccactcctccacctttgacg
hGAPDH-R	5'-catgaggtccaccaccctgt
pch-2-1F	5'-ggaagccaatttcgtctgtc
pch-2-1R	5'-ccccatctctgagttcacaag
sir-2.1-F	5'-tccgacagcaatgttcgata
sir-2.1-R	5'-tttcgaagaagacgaccagaa
act-1-F	5'-tgctgatcgtatgcagaagg
act-1-R	5'-tagatcctccgatccagacg

Western Blot

Cell plates were washed with PBS twice and scripted with RIPA buffer (Boston BioProducts, Ashland, MA) with protease inhibitor cocktail (Roche, Indianapolis, IN). The protein supernatants were collected after centrifuging at 10000 rpm for 20 min at 4 °C and measured by using PierceBCA Protein Assay Kit (Thermo scientific, Rockford, CA). Protein samples (30ug) were subjected to 10% SDS-PAGE and transferred to a polyvinylidene fluoride (PVDF)-membrane (Bio-Rad). The membrane was blocked with 5% BSA for 1 h at room temperature and was incubated with primary antibodies rabbit anti-TRIP13(~49 kDa) (Abcam, Cambridge, MA); mouse anti-β-Actin (~42 kDa) (Abcam) for overnight at 4 °C. Corresponding HRP-labeled secondary antibody was incubated at room temperature for 1 hr. All signal bands were quantified by ImageJ software.

Supplement Table 1-Pch2 Homologs

Gene Symbol	Gene Accession No.	Species
Pch2	NP_009745	*Saccharomyces cerevisiae*
Pch-2	NP_495711	*Caenorhabditis elegans*
Pch2	NP_001287235	*Drosophila melanogaster*
trip13	NP_956876	*Danio rerio*
Trip13	NP_081458	*Mus musculus*
Trip13	NP_001011930	*Rattus norvegicus*
TRIP13	XP_851775	*Canis lupus familiaris*
AT4G24710	NP_194202	*Arabidopsis thaliana*
TRIP13	NP_001159732	*Homo sapiens*

Note: Gene Symbols and Gene Accessible Numbers are from RefSeq database of National Center for Biotechnology Information, NIH (http://www.ncbi.nlm.nih.gov/refseq/).

B

TRIP13 cell line protein over expressed

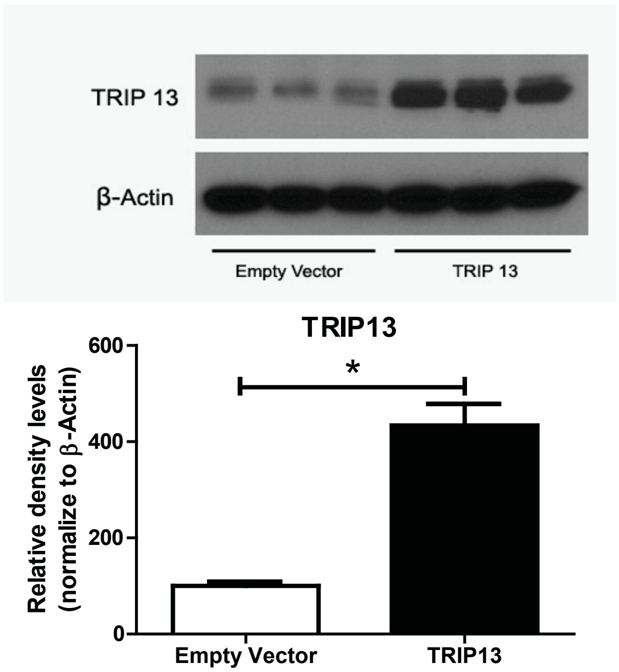

A

TRIP13 cell line mRNA over expressed

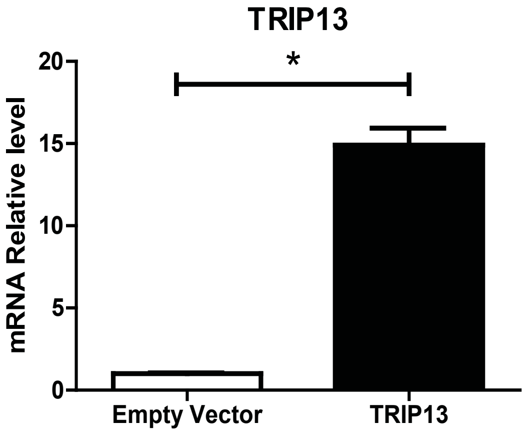

Supplementary Fig.1. Establishment of TRIP13 over-expression Fibroblast cells.
A. mRNA quantification; **B.** protein quantification

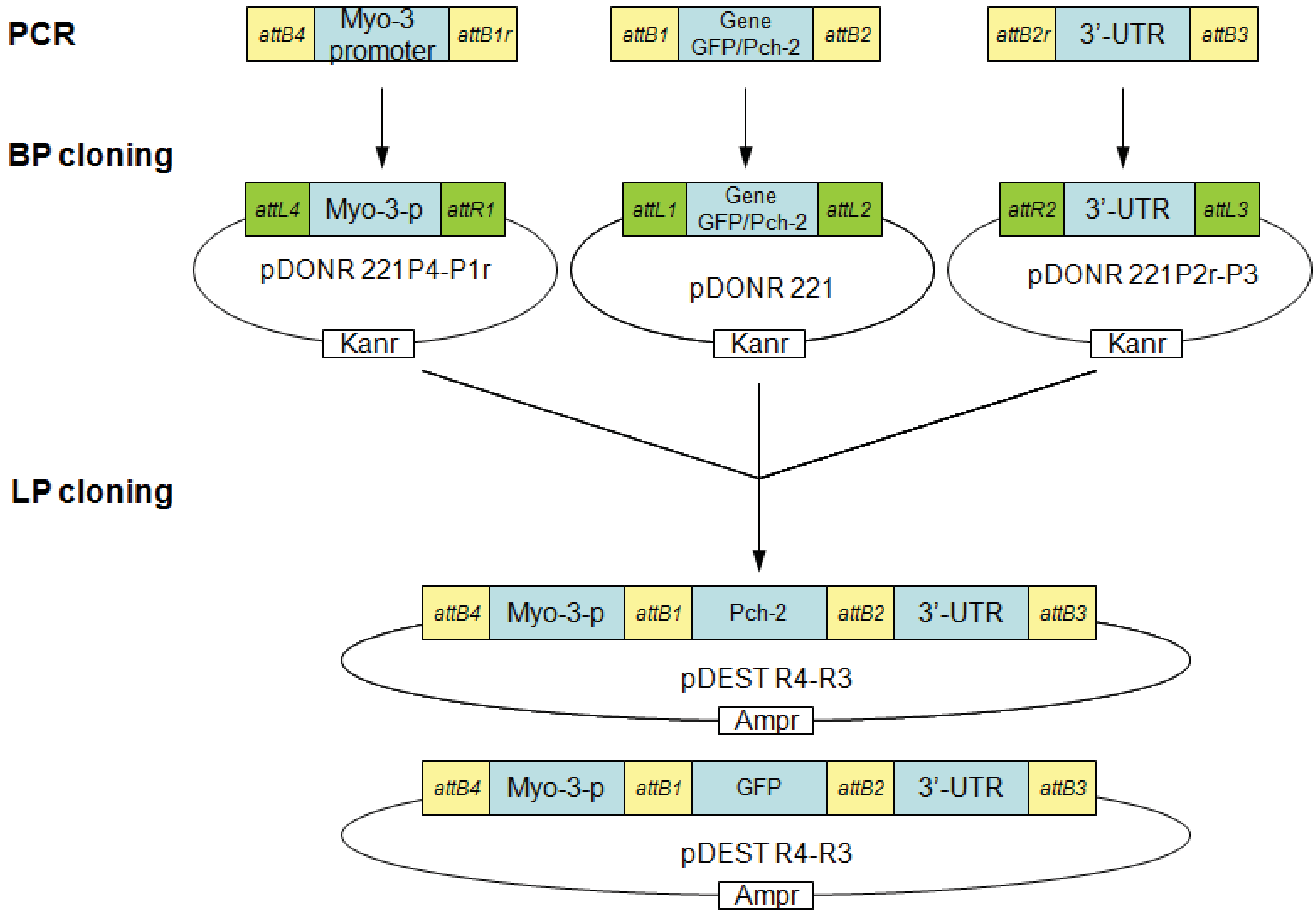

Supplementary Fig.2. Construction of Pch-2 and GFP plasmids.
